# Chronic Exposure to Low-Molecular-Weight Polycyclic Aromatic Hydrocarbons Promotes Lipid Accumulation and Metabolic Inflammation

**DOI:** 10.3390/biom13020196

**Published:** 2023-01-18

**Authors:** Asia Bright, Fenfen Li, Miranda Movahed, Hang Shi, Bingzhong Xue

**Affiliations:** Department of Biology, Georgia State University, Atlanta, GA 30303, USA

**Keywords:** polycyclic aromatic hydrocarbon, white adipocyte, brown adipocyte, macrophage, lipid metabolism, thermogenesis, inflammation, obesity, metabolic disorder

## Abstract

2-naphthol is a low-molecular-weight (LMW) polycyclic aromatic hydrocarbon (PAH) and air pollutant associated with childhood obesity. There has been a recent emergence of studies on the consequences of PAHs on human health. Current epidemiological reports suggest LMW-PAHs may contribute to obesity incidences in children, yet most studies focus on high-molecular-weight PAHs. This study explores 2-naphthol’s impact on obesity and obesity-associated metabolic disorders. To investigate 2-naphthol’s effect on lipid metabolism and inflammation, we employed 3T3-L1 and BAT1 cell lines to model white and brown adipocytes, respectively, alongside a murine macrophage cell line (RAW264.7). We found that 2-naphthol increased the expression of key adipogenic and lipogenic genes while decreasing lipolytic gene expression in chronically treated 3T3-L1 and BAT1 adipocytes. In addition, chronic 2-naphthol treatment also suppressed adrenergic-stimulated thermogenic gene expression in BAT1 brown adipocytes. In consistence, an increase in lipid accumulation was demonstrated in BODIPY and Oil Red O-stained adipocytes. Additionally, 3T3-L1 adipocytes and RAW264.7 macrophages chronically exposed to 2-naphthol showed upregulated mRNA expression of major inflammatory cytokines (e.g., tumor necrosis factor α (*Tnfα),* interleukin-1β *(Il-1β), *and* Il-6)*. In summary, chronic exposure to 2-naphthol stimulates lipid accumulation in adipocytes and inflammation in adipocytes and macrophages. These findings support previous research that demonstrates 2-naphthol has obesogenic potential.

## 1. Introduction

Exposure to polycyclic aromatic hydrocarbons (PAHs) is gaining recognition for their link to the increasing prevalence of metabolic dysfunction [1]. PAHs are a class of heterocyclic xenobiotics compounds produced as byproducts of burning organic matter. They are categorized based on the number of fused aromatic rings in the parent structures. There are two general classifications of PAHs: (1) low-molecular-weight (LMW) PAHs have 2 to 3 aromatic rings, and (2) high-molecular-weight (HMW) PAHs with 4 or more aromatic rings. PAH parent compounds are generally non-reactive, and most adverse health effects can be attributed to their metabolically modified derivatives [2,3]. PAH toxicity and environmental deposition are influenced by physiochemical factors such as structure, molecular weight, and solubility [4,5]. HMW-PAHs usually exist bound to particulate matter, whereas LMW-PAHs remain in the atmosphere as vapors [6,7]. The intrinsic properties of LMW-PAHs make them more pervasive than HMW-PAHs, as lipophilic LMW-PAH metabolites tend to accumulate in fatty tissues (e.g., adipose, liver, and kidneys). 

Most PAHs enter the environment as atmospheric pollution from burning organic matter. Subsequently, the primary route of human exposure is inhalation [8,9]. Exposure to anthropogenic LMW-PAH sources (automobile exhaust, cigarette smoke, and industrial emissions) is rising in tandem with urban and industrial advancements; however, many studies focus on the obesogenic effects of PAH mixtures or HMW-PAHs. Interestingly, Scinicariello and Buser [10] discovered a connection between urinary naphthalene and childhood obesity. Naphthalene is structurally the simplest PAH (made up of only two benzene rings). Using urinary naphthalene metabolites as a biomarker of PAH exposure, increasing concentrations of individual naphthalene metabolites were correlated with body mass index (BMI) and waist circumference rises amongst children [10]. 2-naphthol (2-NAP), a naphthalene metabolite, was the most concentrated LMW-PAH in urine samples, and it showed positive associations with characteristics of childhood obesity [10]. Because we cannot solely rely on correlative studies as sole predictors of illness, exposure-based mechanistic studies are needed to establish causation. Limited research alongside 2-NAP’s biochemical properties hints toward its potential to influence metabolic dysfunction through immunomodulatory mechanisms. For example, studies have shown that mice exposed to a PAH mixture in utero weighed more and had increased adiposity compared to control mice including the increased expression of adipogenic and lipogenic genes in adipose tissues [11]. HMW-PAHs, benzo(a)pyrene and 1-nitropyrene, increase the expression of the proinflammatory cytokines *Il-8*, interferon γ (*Ifnγ*), and Il-1β in lung epithelial cells [12] and splenocytes [13]. Individual HMW-PAHs can also upregulate C-C motif cytokine ligand 1 (*Ccl1*) expression [14] alongside mRNA and protein TNFα levels in cultured human macrophage cells [15]. Additionally, activated macrophages treated with individual PAHs showed increased antigen presentation [16] and IL-1 production proportionate to lipopolysaccharide stimulation [17]. Evidence of individual PAHs activating inflammatory pathways combined with increased associations between LMW-PAHs and obesity warrants further investigation into the impact of 2-NAP on inflammation and lipid metabolism. 

Thus, this research aimed to (1) investigate the role of the LMW-PAH 2-NAP on adipocyte function and lipid metabolism; and (2) determine the impact of 2-NAP exposure on inflammation. The findings of this study may lead to further exploration into the cellular pathways targeted by LMW-PAHs, increasing our understanding of how individual PAHs contribute to metabolic dysfunction.

## 2. Materials and Methods

### 2.1. Cell Culture

RAW264.7 murine macrophages (#TIB-71, ATCC, Manassas, VA, USA) were cultured in Dulbecco’s Modified Eagle’s Medium (DMEM, #11965-092, Gibco/ThermoFisher Scientific, Waltham, MA, USA) containing 10% heat-inactivated fetal bovine serum (FBS, #16000-044, Gibco/ThermoFisher Scientific) and 1% penicillin/streptomycin (P/S, #30-002-CI, CORNING, Corning, NY, USA). Cells were incubated at 37 °C in an atmosphere of 5% CO_2_.

3T3-L1 murine preadipocytes (#CL-173, ATCC) were cultured in an expansion medium (EM) containing DMEM supplemented with 10% calf serum (CS, #16010-159, Gibco/ThermoFisher Scientific) and 1% penicillin/streptomycin (P/S) and incubated as described above. Once preadipocytes reached confluency, cells were cultured for 48 h in differentiation media (DM), containing the growth media (DMEM + 10% FBS + 1% P/S) supplemented with 400 nM insulin (#I6634, Sigma-Aldrich, St. Louis, MO, USA), 0.5 mM 3-isobutyl-1-methylxanthine (IBMX, #I7018, Sigma-Aldrich), and 1 μM dexamethasone (DEX, #D4902, Sigma-Aldrich). Cell culture media was then replaced with maintenance medium (MM), containing the growth media (DMEM + 10% FBS + 1% P/S) supplemented with 400 nM insulin for 2 additional days following differentiation. On day 5, culture media was replaced with MM every 2 days until 3T3-L1 cells reached maturity on day 8.

BAT1 murine preadipocyte (a generous gift from Dr. Patrick Seale, University of Pennsylvania) cultures were grown in a BAT1 expansion medium (BEM) containing DMEM/Nutrient Mixture F-12 (DMEM/F12, #11330-032, Gibco/ThermoFisher Scientific) supplemented with 10% FBS and 1% P/S for 2 days and incubated as described above. Once cells reached 90% confluency, the BEM was replaced with a BAT1 differentiation media (BDM), containing BEM supplemented with 20 nM insulin, 0.5 mM IBMX, 1 μM DEX, 125 μM indomethacin (#I8280, Sigma-Aldrich, and 1 nM triiodothyronine (T_3,_ #T5516, Sigma-Aldrich) for 2 days. After the 2-day differentiation period, the media was replaced with BAT1 maintenance medium (BMM), containing BEM supplemented with 20 nM insulin and 1 nM T_3_, every 2 days until day 8. 

### 2.2. RNA Extraction and RT-PCR Analysis

Cellular RNA was extracted from cultures using the Tri Reagent kit (#RT 111, Molecular Research Center, Cincinnati, OH, USA), as described in the manufacturer’s protocol for cells grown in monolayer. ABI Universal PCR Master Mix and TaqMan primer and probe pair reagents were acquired from Applied Biosystems/ThermoFisher Scientific. Gene expression was evaluated using quantitative RT-PCR using Applied Biosystems QuantStudio 3 real-time PCR system (Applied Biosystems/ThermoFisher Scientific). Target mRNA expression levels were normalized to the expression of the housekeeping gene, cyclophilin, relative to controls. We used cyclophilin primer and probe sequences: 5′-GGTGGAGAGCACCAAGACAGA-3′(forward), 5′-GCCGGAGTCGACAATGATG-3′(reverse), and 5′-TCCTTCAGTGGCTTGTCCCGGCT-3′(probe). All other gene expression primers and probes were purchased from Applied Biosystems. Corresponding cycle threshold (Ct) values were measured, and relative mRNA level was determined using the 2^(−ΔΔCt)^ method.

### 2.3. Enzyme-Linked Immunosorbent Assays (ELISA)

RAW264.7 macrophages were cultured as described above. TNF-α protein production in the cell culture medium was measured using the Mouse TNF-alpha Quantikine ELISA Kit (#MTA00B, R&D Systems™, Minneapolis, MN, USA) according to the manufacturer’s protocol. 

### 2.4. Staining and Visualization of Neutral Lipids

Oil Red O (ORO, #O9755Sigma-Aldrich) staining was employed to visualize lipid accumulation during 3T3-L1 adipogenesis. In preparation for staining, differentiated 3T3-L1 cells were cultured in 6-well plates, washed with PBS, and fixed with 10% formalin overnight. After fixation, cells were washed twice with MilliQ ultrapure water (Sigma-Aldrich) and incubated in a 0.5% ORO solution prepared in isopropanol for 10 min at 37 °C. Plates were rinsed 4 times with ddH_2_O, air-dried, and photographed directly.

To visualize intracellular lipid droplets, triglycerides were labeled with 4,4-difluoro-1,3,5,7,8-pentamethyl-4-bora-3a,4a-diaza-s-indacene (BODIPY 493/503, #D3922, Thermo Fisher Scientific) as previously described [18]. 3T3-L1 adipocytes were seeded on 12 mm collagen-treated glass coverslips at a density of 5 × 10^4^ cells/well in 24-well plates. After differentiation and treatment, as indicated under the figures, cells were fixed in 4% paraformaldehyde (#P6148, Sigma-Aldrich) for 30 min at room temperature. Cells were then incubated in a 2 μM BODIPY solution in the dark for 15 min at 37 °C and washed twice with PBS to remove the residual stain. Coverslips were mounted onto glass slides with ProLong Diamond Antifade Mountant (#P36961, ThermoFisher Scientific). Slides were cured overnight in the ProLong Diamond Antifade Mountant, imaged, and immediately stored at 4 °C. Images of lipid droplets were captured at excitation/emission wavelengths 493/503 nm using an Olympus DP73 photomicroscope and CellSens software (version 1.6) (Olympus, Waltham, MA, USA).

### 2.5. Statistical Analysis

Reported data are expressed as the mean ± SEM. Differences between treatment groups were analyzed for statistical significance by one-way or two-way analysis of variance (ANOVA) with Dunnett’s multiple comparisons test post hoc (GraphPad Prism version 9 (GraphPad Software, La Jolla, CA, USA, www.graphpad.com, access on 27 June 2019). Significantly different group means (*p* < 0.05, indicated by an asterisk *) were calculated using GraphPad Prism version 9 (GraphPad Software, La Jolla, CA, USA, www.graphpad.com, accessed on 27 June 2019).

## 3. Results

### 3.1. In Vitro Effects of Varying LPS Concentrations on RAW264.7 Macrophages

**Figure 1:**
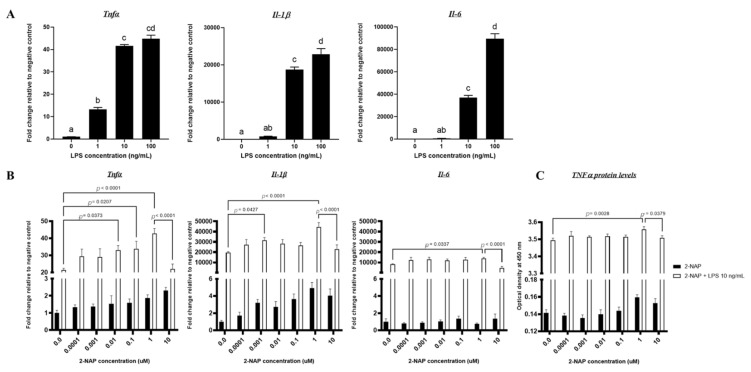
Acute 2-NAP treatment of RAW264.7 macrophages upregulates proinflammatory cytokine expression. (**A**) The effect of LPS on proinflammatory gene expression in RAW264.7 macrophages. Cells were stimulated with LPS for 4 h. Following stimulation, mRNA was extracted and assayed for relative gene expression of *Tnfα*, *Il-1β*, and *Il-6* by RT-PCR. The values shown represent the mean ± SEM of replicate samples from one experiment (*n* = 4). Groups labeled with different letters (a, b, c, and d) are statistically different from each other. (**B**) Macrophages were either treated with 2-NAP alone–six 10-fold concentrations (10 pM–10 μM concentrations) or stimulated with LPS (10 ng/mL) alongside 2-NAP treatment. LPS was used to mimic chronic inflammatory (M1-like) conditions. Solvent control—DMEM + 0.01% ethanol (EtOH); LPS control—DMEM + LPS 10 ng/mL. Upon experimental termination, mRNA was extracted and relative gene expression of *Tnfα*, *Il-1β*, and *Il-6* mRNA was then determined by RT-PCR (*n* = 4). The values shown represent the mean ± SEM. A *p*-value of *p* < 0.05 indicates statistical significance between two groups. (**C**) Protein expression of TNF-α extracted from cellular supernatants (*n* = 4). The optical density of each sample represents readings of TNFα levels secreted into the medium by ELISA assay and was measured using a microplate reader set to 450 nm. Wavelength correction was set to 570 nm. The values shown represent the mean ± SEM. A *p*-value of *p* < 0.05 indicates statistical significance between two groups.

Lipopolysaccharide (LPS) is commonly used to stimulate proinflammatory activation of macrophages in vitro [19]. Proinflammatory cytokines TNFα, IL-1β, and IL-6, are produced by macrophages and adipocytes, the primary cells involved in obesity and metabolic disorders. Given that these cytokines are elevated under obese conditions and promote chronic low-grade inflammation in adipose tissue [20], we measured *Tnfα, Il-1β, and Il-6* mRNA transcript levels to assess the consequences of LPS stimulation in macrophages., RAW264.7 cells were stimulated in vitro across a range of LPS concentrations, from 1 ng/mL to 100 ng/mL, to establish an optimal dose of LPS. As shown in Figure 1A, *Tnfα* and *Il-1β* mRNA levels displayed dose-dependent responsiveness upon LPS stimulation that peaked at a concentration of 10 ng/mL, whereas the expression of *Il*-6 was significantly increased across all concentrations (up to the highest LPS dose—100 ng/mL). Given that PAHs can also activate macrophages and drive proinflammatory responses, the 10 ng/mL LPS concentration was chosen to prevent saturating the proinflammatory responses of RAW264.7 cells. Thus, the LPS dosage of 10 ng/mL was used for subsequent experiments throughout this study.

### 3.2. Acute 2-NAP Treatment Enhances Inflammatory Responses in RAW264.7 Macrophages

Macrophage activation is an essential feature of metabolic inflammation. To assess the effect of 2-NAP on macrophage inflammation, we measured cytokine gene expression in RAW264.7 cells across a wide range of 2-NAP concentrations (six 10-fold concentrations, 0.0001 μM–10 μM) in the absence or presence of 10 ng/mL LPS for 4 h. Results showed that acute 4-hour 2-NAP treatment in RAW 264.7 macrophages had apparent effects on basal *Tnfα, Il-1β,* and *Il-6* expression (Figure 1B). LPS significantly stimulated the expression of these genes in RAW 264.7 macrophages; 2-NAP further enhanced LPS’ effect, with the largest expression enhancement occurring at a 2-NAP concentration of 1 µM (Figure 1B). Furthermore, ELISA analysis of supernatants of LPS-activated RAW264.7 cells treated across the same 2-NAP range revealed that 2-NAP increased basal TNFα levels and significantly enhanced LPS-stimulated TNFα secretion at a 2-NAP concentration of 1 µM level (Figure 1C). We also found that 10 µM 2-NAP treatment reduced Tnfα, Il-β, and Il-6 expression, as well as TNFα secretion as compared to that of 1 µM 2-NAP (Figure 1B,C); however, this may be due to 2-NAP’s toxicity at high doses as it was found that 10 µM 2-NAP reduced adult survival rate in *Daphia magna*, a model system commonly used in toxicological studies, during chronic treatment (Asia Bright, unpublished results). Thus, 1 µM 2-NAP was used in the subsequent studies.

### 3.3. Chronic 2-Naphthol Exposure Increases the Expression of Inflammatory Genes in Macrophages

**Figure 2:**
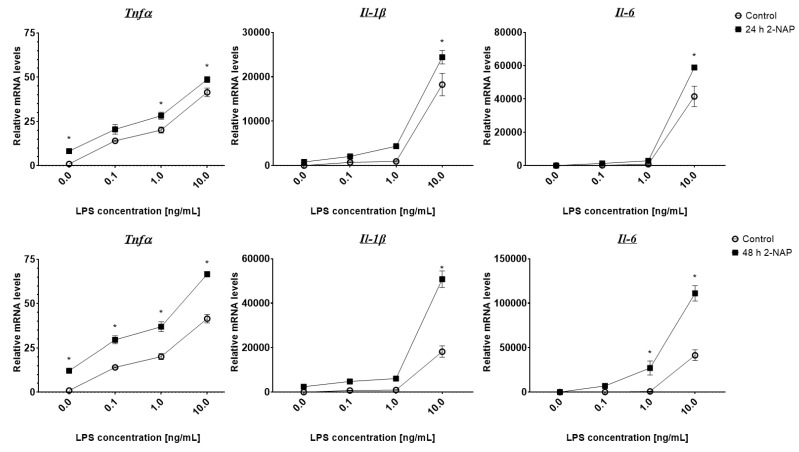
Chronic 2-NAP treatment of RAW264.7 macrophages upregulates mRNA expression of proinflammatory cytokines. (**A**,**B**) Relative mRNA expression levels of proinflammatory markers (*Tnfα*, *Il-1β*, and *Il-6*) in cells treated with 2-NAP (1 µM) for 24 (**A**) and 48 (**B**) hours. RAW264.7 macrophages were first treated with or without 1 µM 2-NAP for 24 (**A**) or 48 (**B**) hours, and then challenged with different concentrations of LPS as indicated for 4 h. Upon experimental termination, mRNA was extracted and relative gene expression of *Tnfα*, *Il-1β*, and *Il-6* mRNA was then determined by RT-PCR (*n* = 4). The values shown represent the mean ± SEM. * Denotes *p* < 0.05 when compared with controls.

Cytokine mRNA expression was measured in RAW264.7 macrophages cultured under chronic 2-NAP exposure conditions (Figure 2). RAW264.7 macrophages were treated with 1 µM 2-NAP for either 24 or 48 h, then challenged with various concentrations of LPS for 4 h. We found that chronic 2-NAP treatment for 24 (Figure 2A) or 48 (Figure 2B) hours significantly increased basal *Tnfα* expression and significantly enhanced LPS-induced inflammatory gene expression, including *Tnfα*, *Il-1β,* and *Il-6* (Figure 2).

### 3.4. Chronic 2-Naphthol Treatment Up-Regulates An Adipogenic and Lipogenic Program Gene Expression in 3T3-L1 Cells

**Figure 3:**
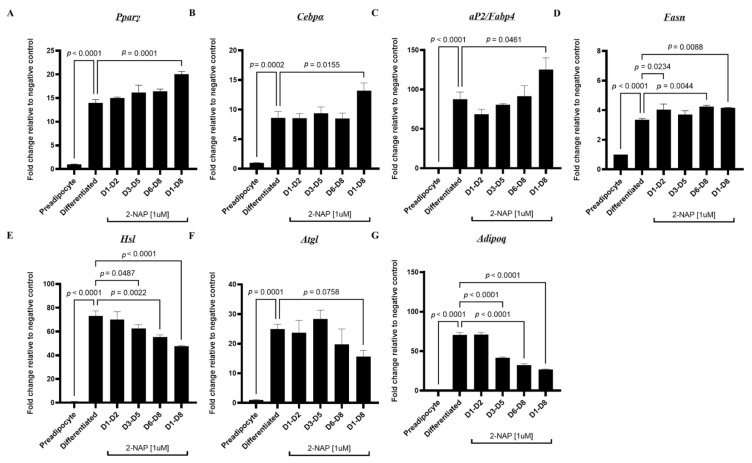
Chronic 2-naphthol treatment regulates adipogenic, lipogenic, and lipolytic gene expression in 3T3-L1 adipocytes. Relative mRNA expression of genes encoding for adipogenic transcription factors (**A**) *Pparγ* and (**B**) *Cebpα*; adipocyte-specific marker (**C**) ap2/Fabp4, lipogenic and lipolytic markers (**D**) Fasn(**E**) *Hsl* and (**F**) *Atgl*; and the adipokine (**G**) *Adipoq* was measured in preadipocytes and differentiated 3T3-L1 adipocytes treated with 1 µM 2-NAP at different differentiation stages as indicated. Upon experimental termination, mRNA was extracted and relative gene expression was then determined by RT-PCR (*n* = 4). The values shown represent the mean ± SEM. A *p*-value of *p* < 0.05 indicates statistical significance between two groups.

Next, we sought to investigate the effects of 2-NAP on adipogenesis and lipid metabolism. 3T3-L1 murine preadipocytes were induced to differentiate with a differentiation medium and were concurrently treated with either PBS or 2-NAP on days 1–2, 3–5, 6–8, or 1–8 of differentiation. Cells from all treatment groups were harvested on day 8 of differentiation. Adipocytes exposed to 2-NAP during differentiation show an overall increase in adipogenesis (Figure 3). More specifically, chronic 2-naphthol treatment, especially during the entire 1–8 days of differentiation, upregulated 3T3-L1 adipogenesis, as shown by increased mRNA expression of adipogenic markers such as peroxisome proliferator-activated receptor gamma (*Pparγ*), CCAAT enhancer binding protein α (*Cebpα*), and adipocyte protein 2/fatty acid binding protein 4 (*aP2/Fabp4*) (Figure 3). Additionally, chronic 2-NAP treatment from days 1–8 increased the expression of a key lipogenic enzyme, fatty acid synthase (*Fasn*), while coordinately downregulating genes involved in lipolysis, including the significant downregulation of hormone-sensitive lipase (*Hsl*) and the tendency of downregulation of patatin-like phospholipase domain containing 2/adipocyte triglyceride lipase (*Pnpla2/Atgl*) (Figure 3). Moreover, chronic 2-NAP treatment during differentiation significantly decreased the adipokine adiponectin (*Adipoq*) expression (Figure 3). In 3T3-L1 cells treated with 2-NAP during the differentiation days 6–8, there was also an increase in *Fasn* expression and a coordinated decrease in *Hsl* expression (Figure 3). Thus, our data suggest that chronic 2-NAP treatment in adipocytes may promote an adipogenic and lipogenic program by coordinated regulation of gene expression involved in adipogenesis, lipogenesis, and lipolysis.

### 3.5. Qualitative Analysis Shows An Increase in Lipid Accumulation in 2-NAP Treated 3T3-L1 Adipocytes

**Figure 4:**
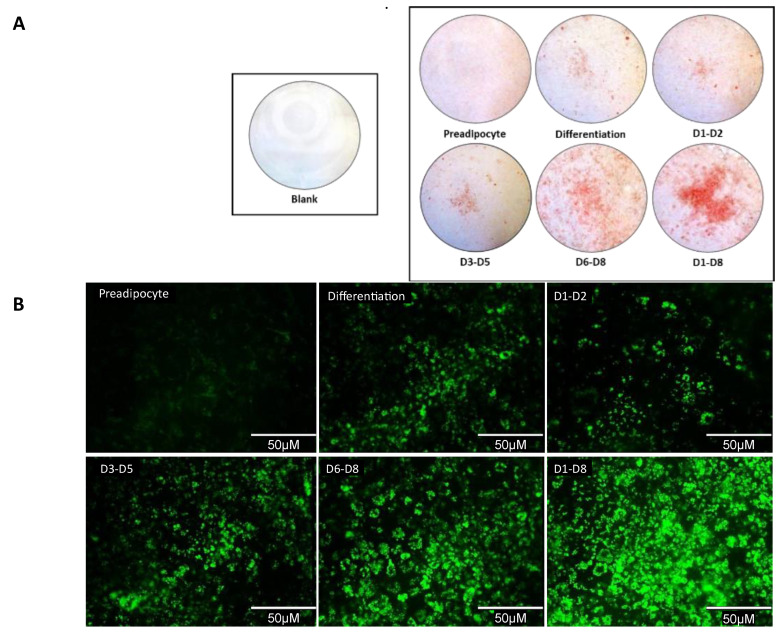
Phenotypic analysis of 2-naphthol’s effect on adipocyte differentiation and lipid accumulation by oil red O and BODIPY staining. Upon experimental termination, 3T3-L1 cells were fixed, stained, and photographed either directly (whole-well view) or at ×40 magnification (microscopic view). (**A**) Whole-well imaging comparing ORO (red) stained 3T3-L1 preadipocytes differentiated under chronic 2-NAP treatments versus their controls. (**B**) Confocal microscopic images of BODIPY 493/503 (green) stained neutral lipid content in 3T3-L1 cells under chronic 2-NAP treatment. The scale bar spans 50 μM.

We then examined lipid accumulation in 3T3-L1 adipocytes treated with 2-NAP by either oil red O (ORO) or BODIPY staining. 3T3-L1 cells were photographed directly for a whole-well view (Figure 4A) or at 40× magnification for a detailed microscopic view (Figure 4B). Consistent with results in Figure 3, both ORO and BODIPY staining revealed increased lipid accumulation in adipocytes under chronic 2-NAP treatment from days 1–8 and 6–8 (Figure 4). Adipocytes treated during the early and middle stages of differentiation (days 1–2 and 3–5) did not show a measurable increase in lipid accumulation compared to the differentiation control (Figure 4).

### 3.6. Chronic 2-NAP Exposure Increases the Expression of Inflammatory Genes in 3T3-L1 Cells 

**Figure 5:**
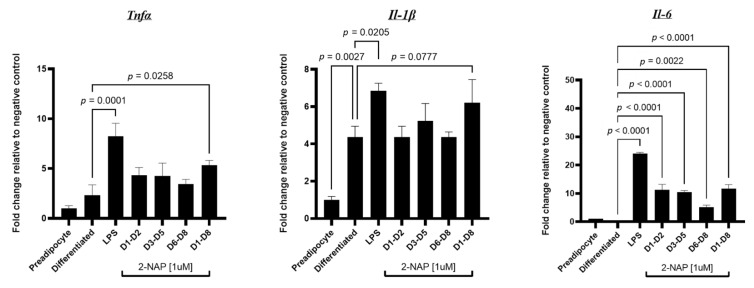
Chronic 2-NAP exposure increases the expression of inflammatory genes in 3T3-L1 cells. Relative mRNA expression of inflammatory genes (*Tnfα*, *Il-1β*, and *Il-6*) in 3T3-L1 adipocytes chronically treated with 1 µM 2-NAP as indicated. LPS (1 ng/mL) was used as a positive control. Upon experimental termination, mRNA was extracted and relative gene expression was then determined by RT-PCR (*n* = 4). The values shown represent the mean ± SEM. A *p*-value of *p* < 0.05 indicates statistical significance between two groups.

To determine whether chronic 2-NAP treatment in 3T3-L1 cells contributes to adipocyte inflammation, we treated 3T3-L1 cells with 1 µM 2-NAP at specific points during differentiation, from differentiation days 1–2, 3–5, 6–8, and 1–8. Cells were harvested on differentiation day 8 for gene expression analysis. For the positive control LPS treatment, 3T3-L1 cells were differentiated without 2-NAP treatment and treated with 10 ng/mL LPS for 4 h on differentiation day 8 before harvesting the cells. As expected, 3T3-L1 adipocytes treated with LPS significantly induced inflammatory gene expression, including *Tnfα*, *Il-1β,* and *Il-6* (Figure 5). Interestingly, 3T3-L1 adipocytes chronically exposed to 2-NAP also showed a significant increase in mRNA expression of proinflammatory cytokine markers *Tnfα* and *Il-6* and a tendency for increased *Il-1β* expression (Figure 5). 

### 3.7. Brown Adipocytes Exposed to 2-NAP during Differentiation Displayed A Decrease in Thermogenesis and An Increase in Adipogenic Gene Expression

**Figure 6:**
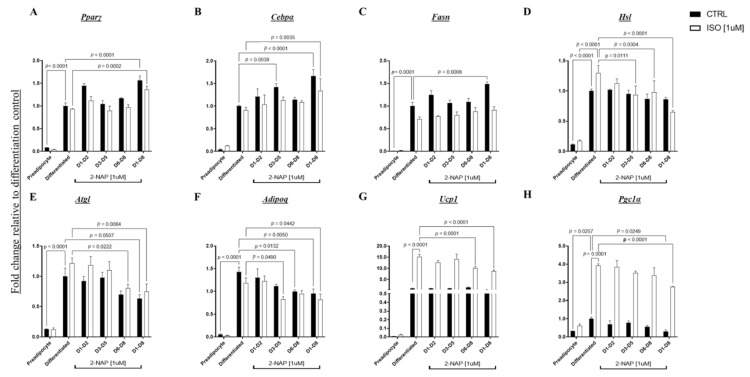
Effect of 2-NAP on thermogenic and adipogenic genes in BAT1 cells. Relative mRNA expression of adipogenic transcription factors (**A**) *Pparγ* and (**B**) *Cebpα*; markers of lipogenesis and lipolysis (**C**) *Fasn*; (**D**) *Hsl*, and (**E**) *Atgl*; the adipokine (**F**) *Adipoq*; and thermogenic genes (**G**) *Ucp1* and (**H**) *Pgc1α* was measured in BAT1 brown adipocytes chronically treated with 1 µM 2-NAP as indicated. On day 8, half of the cells were further treated with 1 µM isoproterenol (Iso) for 4 h. Upon experimental termination, mRNA was extracted and relative gene expression was then determined by RT-PCR (*n* = 4). The values shown represent the mean ± SEM. A *p*-value of *p* < 0.05 indicates statistical significance between two groups.

To determine the role of LMW-PAH exposure in the regulation of thermogenesis and brown-fat-specific gene expression, BAT1 cells were chronically exposed to 2-NAP at different points of differentiation from days 1–2, 3–5, 6–8, and 1–8. On day 8 of differentiation, BAT1 cells were treated with 1 µM of the β-adrenergic agonist isoproterenol (ISO) for 4 h before cells were harvested for gene expression analysis. As expected, differentiated BAT1 brown adipocytes exhibited significantly increased expression of genes in adipocyte differentiation and thermogenesis compared to that of preadipocytes, including markers of adipocyte differentiation *Pparγ* and *Cebpα*, lipogenesis marker *Fasn*, lipolysis markers *Hsl* and *Atgl*, adipokine *adipoq*, and thermogenesis markers uncoupling protein 1 (*Ucp1*) and PPARG coactivator 1 alpha (*Pgc1α*) (Figure 6). ISO-treated BAT1 cells showed increased expression of thermogenic and lipolytic markers *Ucp1, Pgc1α,* and *Hsl* (Figure 6). Similar to 3T3-L1 cells, BAT1 cells treated with 2-NAP across the entire cellular differentiation process (days 1–8) showed increased expression of adipogenesis genes, including the adipocyte differentiation markers *Pparγ* and *Cebpα* and the lipogenesis marker *Fasn*. 2-NAP treatment during BAT1 differentiation resulted in significant downregulation of *adipoq* (Figure 6). BAT1 brown adipocytes treated with 2-NAP during days 1–8 of differentiation resulted in a downregulation in basal expression of *Pgc1α* and a tendency of downregulation in basal expression of *Atgl*. Furthermore, chronic 2-NAP treatment during BAT1 cell differentiation significantly downregulated ISO-stimulated *Ucp1*, *Pgc1α*, *Hsl*, and *Atgl* expression (Figure 6). 

## 4. Discussion

We are exposed daily to polycyclic aromatic hydrocarbons (PAH). Researchers have previously discovered associations between the presence of PAH metabolites in our body and the incidence of obesity and obesity-related comorbidities [10,21,22]. Most studies focus on HMW-PAH; however, little is known about the effects of individual LMW-PAHs such as naphthalene, which is more pervasive and easier metabolized into bioactive compounds (1-naphthol and 2-naphthol). In vivo and in vitro studies have revealed the obesogenic potential LMW-PAHs such as 2-NAP [9,23]. On the basis of this previous research, we aimed to identify the cellular pathways LMW-PAHs may influence, contributing to metabolic dysfunction associated with obesity. This work investigated how 2-NAP exposure impacts adipocyte and macrophage physiologies using RAW264.7, 3T3-L1, and BAT1 murine cell lines to model key regulators of adipose tissue health. These in vitro models represent macrophages, white adipocytes, and brown adipocytes, respectively. These models allowed us to analyze the effect of 2-NAP on the overall inflammatory status and at distinct stages of adipocyte differentiation. Using these models, we investigated (1) the role of 2-NAP on adipocyte function and lipid metabolism; and (2) the impact of 2-NAP exposure on adipose inflammation. 

Adipogenesis, the process by which new adipocytes are generated, is defined by sequences of cellular recruitment, expansion, differentiation, and maturation of committed preadipocytes [24]. Distinct transcriptional events mark these developmental stages. Key transcription factors, *Pparγ* and *Cebpα,* are upregulated during early differentiation. This research reveals that *Pparγ* and *Cebpα* mRNA are significantly increased in white adipose and brown adipose tissues chronically exposed to 2-NAP (Figure 3 and Figure 6.) The stimulation of these transcription factors activates a battery of other target genes during late differentiation, including *aP2*, a marker specific to mature adipocytes, lipogenic marker *Fasn*, lipolysis markers *Hsl* and *Atgl*, and the adipokine *adipoq*. Interestingly, we found that 2-NAP treatment during 3T3-L1 white adipocyte differentiation and BAT1 brown adipocyte differentiation significantly stimulated the expression of *Pparγ* and *Cebpα*. In addition, 2-NAP treatment significantly increased the expression of *Fasn*, an important enzyme involved in lipogenesis, and coordinately downregulated the expression of *Hsl* and *Atgl*, enzymes involved in lipolysis. This coordinated regulation in adipogenic, lipogenic, and lipolytic gene expression could contribute to the increased lipid accumulation observed in 2-NAP-treated adipocytes (Figure 4), which supports our hypothesis of 2-NAP as a potential obesogen. Interestingly, we found that continuous 2-NAP presence rather than transient treatment during the differentiation process may be required for 2-NAP’s obesogenic function, as transient treatment of 2-NAP during the early and mid-stage of differentiation did not enhance lipid accumulation in either 3T3-L1 white adipocytes or BAT1 brown adipocytes. 

The observed decrease in *adipoq* mRNA expression aligns with previous work showing decreased adipoq mRNA expression in *ob/ob* mice compared to lean ob/+ mice [25]. Furthermore, serum adiponectin levels in wild-type mice on a high-fat diet or *db/db* mice are decreased compared with control animals [26]. Similar trends of *adipoq* dysregulation were discovered upon examination of the plasma from obese humans [27]. Adiponectin has been shown to contribute to a beneficial metabolic phenotype through various functions, including suppressing hepatic glucose production, stimulating fatty acid oxidation in muscle, and inhibiting inflammation in various tissues [28,29]. Thus, inhibiting adiponectin (*adipoq*) expression in both 3T3-L1 white adipocytes and BAT1 brown adipocytes using 2-NAP may contribute to the impact of 2-NAPon metabolic dysregulation. 

While the function of white adipocytes is to store excess energy as fat, that of brown adipocytes is to dissipate energy as heat through adaptive thermogenesis due to its unique expression of *Ucp1* in the mitochondria inner membrane [30,31]. UCP1 acts to uncouple oxidative phosphorylation from ATP synthesis, dissipating energy as heat and profoundly increasing overall energy expenditure [32,33]. Brown adipocyte thermogenesis is regulated by catecholamine signaling. Catecholamines released by sympathetic nerve terminals in response to cold stimulate lipolysis and activate BAT/beige thermogenesis via β-adrenergic receptors [34,35]. Our findings showed that 2-NAP significantly suppressed the β-adrenergic receptor agonist isoproterenol-stimulated *Ucp1* expression in BAT1 brown adipocyte cells, which may contribute to its obesogenic potential.

Obesity is associated with a chronic low-grade inflammatory state, and adipose tissue inflammation is causatively linked to obesity-associated metabolic disorders, including insulin resistance and type 2 diabetes [36,37]. An essential feature of obesity-induced inflammation is the infiltration of macrophages into adipose tissue, which, together with inflamed adipocytes, contributes to obesity-associated chronic inflammation [38,39]. We found that chronic 2-NAP treatment in macrophages and adipocytes significantly increased the expression of proinflammatory cytokines. Thus, chronic exposure to the obesogen 2-NAP may further precipitate metabolic disorders by enhancing macrophage and adipocyte inflammation.

It has been well documented that long-term high fat diet (HFD) feeding causes white adipose tissue (WAT) remodeling, as evidenced by enlarged adipocytes, exaggerated inflammation, and increased fibrosis, leading to WAT dysfunction [40,41]. Although little is known about whether LMW PAHs contribute to adipose tissue fibrosis, evidence from the literature suggests a potential link between air pollutant exposure and fibrogenesis and tissue dysfunction. For example, PM2.5 is a type of air pollutant that contains particulate matter with a diameter of 2.5 micrometers or less. Studies have shown that PAHs make up a significant proportion of the chemical concentration in PM2.5 samples from most urban atmospheres [42]. Mice on a high-fat diet exposed to air pollution mixtures via inhalation showed increased levels of collagen type 1 protein and fibrosis development in the liver [43]. In addition, RAW264.7 cells exposed to the same mixture upregulated TGFβ mRNA expression [43]. PM2.5 exposure also induced the onset of a non-alcoholic steatohepatitis-like phenotype in mice [44]. As naphthalene and 2-naphthalene are components of PM2.5 mixtures, they may potentially promote fibrogenesis in inflamed adipose tissue, leading to adipose tissue dysfunction.

Overall, our findings show that 2-NAP significantly enhances adipocyte lipid accumulation by upregulating adipogenic (*Pparγ*, *Cebpα*) and lipogenic (*Fasn*) biomarkers while downregulating markers of lipolysis (*Hsl*, *Atgl*) in a differentiation stage-dependent fashion. In addition, we found that 2-NAP downregulates adiponectin expression in white and brown adipocytes and inhibits ISO-stimulated thermogenic expression in brown adipocytes. Moreover, 2-NAP also significantly stimulates proinflammatory cytokine expression in white adipocytes and macrophages. Data collected from this study will help characterize the role of PAHs in metabolism, obesity, and inflammation. Further characterization of this pathway may have significant implications for developing preventative and therapeutic strategies for many metabolic disorders.

## Data Availability

All datasets will be available upon request to the corresponding authors Hang Shi and Bingzhong Xue.
